# Endophytic Fungi of Salt-Tolerant Plants: Diversity and Ability to Promote Plant Growth

**DOI:** 10.4014/jmb.2106.06007

**Published:** 2021-09-15

**Authors:** Irina Khalmuratova, Doo-Ho Choi, Jong-Guk Kim, In-Seon Lee

**Affiliations:** 1 School of Life Science and Biotechnology, Kyungpook National University, Daegu 41566, Republic of Korea; 2 Department of Food Science and Technology, Keimyung University, Daegu 42601, Republic of Korea

**Keywords:** Coastal plants, fungal diversity, fungal endophytes, plant growth-promoting activity, gibberellin

## Abstract

*Suaeda australis*, *Phragmites australis*, *Suaeda maritima*, *Suaeda glauca* Bunge, and *Limonium tetragonum* in the Seocheon salt marsh on the west coast of the Korean Penincula were sampled in order to identify the endophytes inhabiting the roots. A total of 128 endophytic fungal isolates belonging to 31 different genera were identified using the fungal internal transcribed spacer (ITS) regions and the 5.8S ribosomal RNA gene. *Fusarium*, *Paraconiothyrium* and *Alternaria* were the most commonly isolated genera in the plant root samples. Various diversity indicators were used to assess the diversity of the isolated fungi. Pure cultures containing each of the 128 endophytic fungi, respectively, were tested for the plant growth-promoting abilities of the fungus on Waito-C rice germinals. The culture filtrate of the isolate Lt-1-3-3 significantly increased the growth of shoots compared to the shoots treated with the control. Lt-1-3-3 culture filtrate was analyzed and showed the presence of gibberellins (GA_1_ 2.487 ng/ml, GA_3_ 2.592 ng/ml, GA_9_ 3.998, and GA_24_ 6.191 ng/ml). The culture filtrate from the Lt-1-3-3 fungal isolate produced greater amounts of GA_9_ and GA_24_ than the wild-type *Gibberella fujikuroi*, a fungus known to produce large amounts of gibberellins. By the molecular analysis, fungal isolate Lt-1-3-3 was identified as *Gibberella intermedia*, with 100% similarity.

## Introduction

In their natural state, most plants are colonized by endophytes that can form interactions with their host. This can improve the tolerance to environmental stresses, thus improving the ecological adaptability of host plants. Symbiotic microorganisms such as endophytes are known to play an important role in the regulation of host plant growth and development processes. Stimulation of plant growth by endogenous fungi is due in part to the production of plant hormones such as gibberellins (GAs), abscisic acids, cytokinins, auxins, and other plant growth-promoting substances. Phytohormones may also confer fitness benefits to host plants, including tolerance to herbivores, salinity, diseases, heat, and drought, and may also increase shoot and root biomass [1-6]. Recent studies revealed that symbiotic fungi play a critical role in host plant survival [7, 8].

*Gibberella intermedia* is a well-known fungus isolated from plants and soil and found as a pathogen in many plants worldwide. Mycotoxins, including fusaproliferin, beauvericin, moniliformin, and fumonisin, may also be secreted by strains of *G. intermedia* [9]. Within the same genus, *G. fujikuroi* can produce gibberellins, a group of diterpenoid plant hormones that were first detected in the 1930s in a culture filtrate from this fungus. Gibberellins affect plant growth in a variety of ways, including stem growth, germination, flowering, sexual expression, dormancy, enzyme induction, and leaf-and-fruit aging. Recently, more than 136 gibberellins isolated from bacteria, fungi, and plants have been identified. Fungal strains *Penicillium citrinum* LWL4, *Aspergillus terreus* LWL5, *Paecilomyces formosus* LHL10, and *Sphaceloma manihoticola* have been reported to produce gibberellin [10-12].

Recently, interest in the destruction of natural ecosystems, especially salt marshes, has been increasing. To restore the damaged salt marshes, greening marshes by promoting the growth of native plants is essential. In this process, endophytic fungi are the key to restoration. The aims of this study were to: a) survey the distribution and diversity of endophytic fungi from a specific region of Korea, b) assess the ability of the fungal isolates to promote growth of Waito-C rice germinals, and c) to determine whether secondary metabolites, such as gibberellins, are present in fungal culture filtrates.

## Materials and Methods

### Collection of Plant Samples

Five plant species were collected from the Seocheon salt marsh, located in the middle of the west coast of Korea. Within each species, two to four samples were collected at different locations at each site. In Table 1, lists of the scientific name, codes of the plants, and global positioning system information were organized. The samples were placed into zipped bags during collection, transported to the laboratory, and stored at 4°C until further processing for isolation of endophytes. All sample preparation was done within 36 h of collection.

### Surface Sterilization and Isolation of Endophytic Fungi from the Roots

Samples were washed with distilled water to remove the soil particles and residues from the plant roots. Washed samples were sterilized with Tween 80 detergent solution for 5 min and surface of samples was sterilized by perchloric acid (1%) solution. The samples were then washed twice with sterile distilled water. The prepared root samples were then cut into 2.0–2.5 cm pieces by using scissors. The pieces were cultured on Hagem minimal medium (glucose 5.0 g/l, malt extract 5.0 g/l, NH_4_Cl 0.5 g/l, KH_2_PO_4_ 0.5 g/l, MgSO_4°_7H_2_O 0.5 g/l, and Fe-EDTA 11.3 mg/l) containing 80 ppm streptomycin [13]. The plates were incubated at 25°C until single colonies of fungi were emerged. As fungi emerged, they were transferred to potato dextrose agar (PDA; potato extract 4 g/l and dextrose 20 g/l) plates for pure cultures.

### DNA Extraction, PCR Amplification, and Identification of Fungal Strains

The fungal isolates were inoculated into Erlenmeyer flasks containing 50 ml of the potato dextrose broth medium and were incubated at 25°C ± 2°C for 7 days on a rotary shaker at 120 rpm. Lyophilized fungal isolates were collected using vacuum. Fungal genomic DNA extracted using the DNeasy Plant Mini Kit (Qiagen, USA) was identified by amplification and sequencing of the internal transcribed spacer (ITS) region. Among the various ITS, ITS primers named ITS-1 (5′-TCC GTA GGT GAA CCT GCG G-3′) and ITS-4 (5′-TCC TCC GCT TAT TGA TAT GC-3′) were used in this study. The reaction cycles comprised an initial denaturation step (95°C for 2 min); 30 cycles of denaturation (95°C for 30 sec), annealing (55°C for 1 min), and extension (72°C for 1 min); and a final extension (72°C for 7 min). Detection of PCR production was processed using agarose gel electrophoresis with ethidium bromide staining. The products were directly purified by the QIAquick PCR Purification Kit (Qiagen). Then, purified PCR products were sequenced by the ABI PRISM BigDye Terminator Cycle Sequencing Ready Reaction Kit (PE Biosystems, USA) and an ABI 310 Genetic Analyzer (Perkin Elmer, USA). The obtained sequences were identified using the National Center for Biotechnology Information GenBank database BLASTN tool.

### Statistical Analysis

Diversity of the fungal endophytes isolated from the plant samples was assessed. Diversity at the genus level was assessed using the Shannon diversity index (*H′*), Fisher’s alpha index (*α*), and Simpson’s index of diversity (*1 - D*). Richness was evaluated using Menhinick’s richness index (*D_mn_*) and Margalef ’s index (*D_mg_*) [14-16].

### Effect of Fungal Filtrates on Plant Growth Promotion on Waito-C Rice Seedlings

To test the ability of the fungal isolates to promote plant growth, Waito-C rice germinals were exposed to culture filtrates from the fungal isolates. The fungal isolates were cultured in Czapek Dox broth medium on a shaking incubator for 7 days at 25°C and 180 rpm and were harvested using 20~25 μm/15 cm Whatman filter papers (Brentford, UK). The harvested fungal culture filtrates were immediately frozen at −70°C and then lyophilized for concentration. The lyophilized culture filtrates were concentrated 50 times by mixing with 1 ml of distilled water. In order to minimize the activity of gibberellins in the seed coat, Waito-C rice grains were treated with uniconazole for 24 h. The treated seeds were washed and soaked in distilled water until radical emergence. The young seedlings were then transplanted to glass tubes with 0.6% water agar medium and the seedlings were grown in a growth chamber under conditions of 12 h at 25°C and 12 h at 30°C for a total of 5 days. After rice seedlings reached the two-leaf stage, 10 microliters of concentrated lyophilized culture filtrate from each fungal isolate was applied to the apical meristem. In order to compare with *G. fujikuroi*, whose performance was previously confirmed, the same amount with same concentrate as in other experimental groups was inoculated into the same site and the experiment was conducted. In this study, plant and shoot lengths of rice became apparent 1 week after application compared to rice seedlings treated with wild-type *G. fujikuroi*, which served as positive controls.

### Extraction and Quantification of Gibberellins from Fungal Culture Filtrates

Fungal isolates were incubated for 7 days in Czapek Dox broth medium with 1% (w/v) glucose and peptone. Gibberellins were extracted from culture filtrates and were analyzed using reverse-phase C_18_ high-performance liquid chromatography. With selected ion monitoring (SIM), fractions were then prepared for gas chromatography (GC) with mass spectrometry (MS). Three major ions of the added [^2^H_2_] gibberellin internal standards and gibberellins were monitored simultaneously. Retention time calculations involved calculating the Kovats retention index using hydrocarbon standards. Gibberellins were quantified using the peak area ratios between un-deuterated and deuterated gibberellins.

## Results and Discussion

### Molecular Identification of Endophytic Fungi

The nucleotide sequences of endophytic fungal isolates from the Seocheon salt marsh were grouped by host plant species and registered in GenBank with the following accession numbers: *S. australis* (KP017930–KP017952), *P. australis* (KP017953–KP017967), *S. maritima* (KP017968–KP017981), *S. glauca* Bunge (KP017982–KP018018), and *L. tetragonum* (KP018019–KP018057). In table 1, the accession numbers of fungal isolates were listed. Total 128 fungal isolates, representing 31 genera and 37 species, were isolated from plants on the west coast of Korea.

The 128 isolates were classified as 2 phyla, Ascomycota and Basidiomycota. The Dothideomycetes class (57 strains) accounted for the greatest number of strains, followed by Sordariomycetes (49 strains), Eurotiomycetes (17 strains), Ustilaginomycetes (3 strains), Agaricomycetes (1 strain), and Leotiomycetes (1 strain). At the genus level, *Fusarium* accounted for the highest number of strains (23 strains), followed by *Paraconiothyrium* (15 strains), and *Alternaria* (14 strains).

**Fig. 1:**
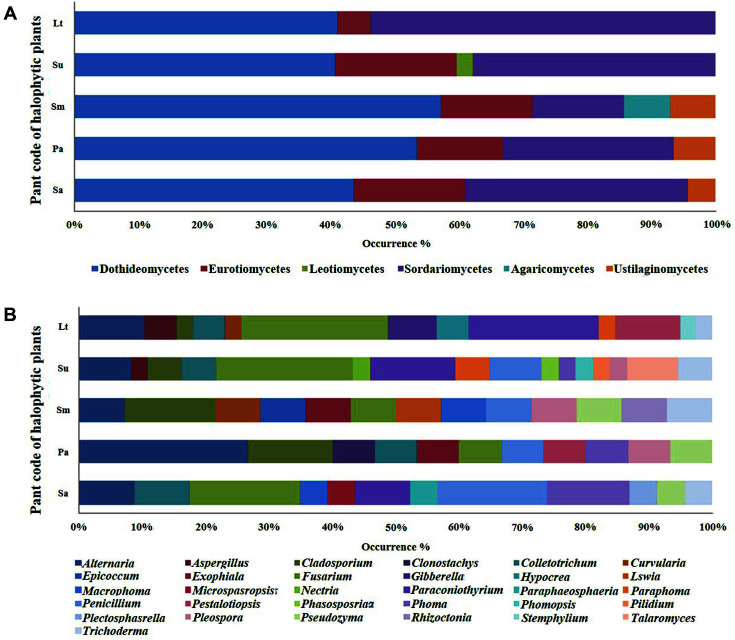
Distribution of fungal isolates in different plant samples at the class (**A**) and genus (**B**) levels. Sa, *Suaeda australis*; Pa, *Phragmites australis*; Sm, *Suaeda maritima*; Su, *Suaeda glauca* Bunge; and Lt, *Limonium tetragonum*.

Endophytic fungi were also analyzed at the class and genus levels (Fig. 1) and Dothideomycetes was found to account for the highest percentage of isolates at the class level. Except for the isolates from the plant *L. tetragonum*, Dothideomycetes accounted for more than a half of all of the fungal isolates in every plant sample. *Fusarium* was the most prevalent (18%) among all of the fungal strains, followed by *Paraconiothyrium* (11.7%), and *Alternaria* (10.9%). The remaining genera of the fungal isolates constituted only 0.7–7% of the strains. A study by Nalini *et al*.(2014) showed that fungi from the *Fusarium* genus are the most salient fungi discovered in stem and root fragments of medicinal plants that are native to the western coast of the Indian peninsula [17]. Fungal species abundant in the host, including fungi from the genera *Fusarium*, *Alternaria*, and *Penicillium* can associate with plants [18, 19].

### Diversity of Endophytic Fungi Isolated from Halophytes

In Table 2, more details of fungal components were organized. Depending on the plants, fungal isolates from *S. australis* were classified as 12 genera and 11 species. Isolates from *P. australis* were classified as 11 genera and 8 species. Isolates from *S. maritima* were classified as 13 genera and 8 species. Isolates from *S. glauca* Bunge were classified as 16 genera and 16 species. Fungal isolates from *L. tetragonum* were classified as 13 genera and 17 species.

The richness and diversity of the fungal isolates were analyzed at the genus level and more detail of fungal information was arranged on the Table 3. For general abundance calculated using Menhinick and Margalef indices, *S. maritima* scored the highest at 3.47 and 4.55, respectively. In terms of genetic diversity, which was calculated using Shannon’s, Fisher’s α, and Simpson’s indices, *S. maritima* had the highest scores of 2.54, 88.78, and 0.99, respectively. The diversity scores vary greatly because the diversity indices are based on the proportional abundance of genera in a sample, and are more sensitive toward genus evenness [20]. The *S. maritima* specimen had the highest diversity indices, indicating the most diverse fungal community isolated from this plant.

### Bioassay for Plant Growth-Promoting Effects of Fungal Culture Filtrates on Waito-C Rice Seedlings

**Fig. 2:**
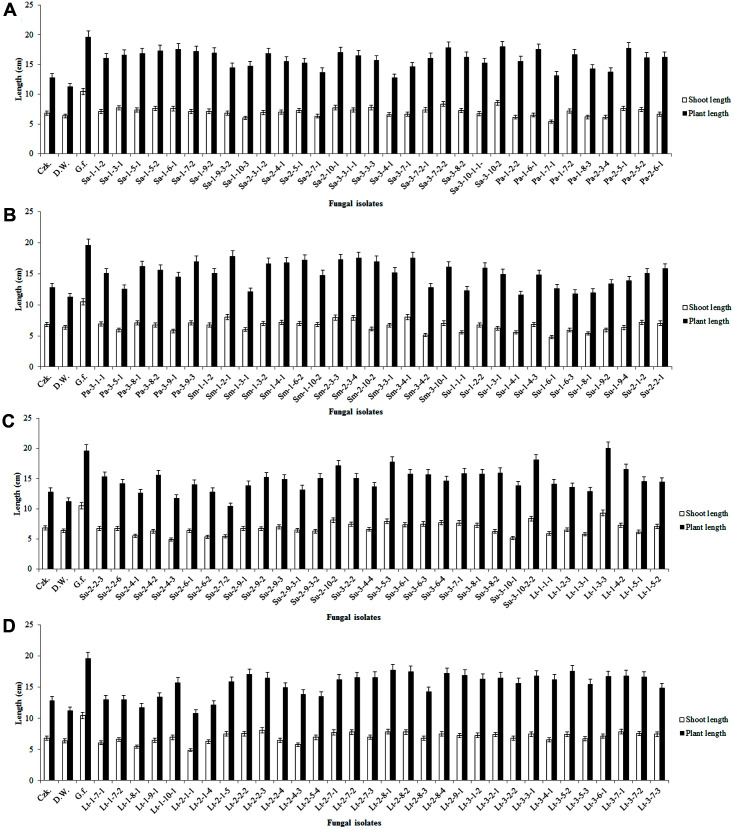
Bioassay for plant growth promoting of Waito-C rice seedlings with culture filtrates of fungi isolated from the plant samples (A-D). Ten microliters of lyophilized culture filtrates was treated to Waito-C rice seedlings. The shoot length and plant length of the Waito-C rice seedlings were measured after 7 days of treatment. The standard deviation from means was calculated using Microsoft Excel. Czk., Czapek Dox broth media; D.W., distilled water; G.f., *Gibberella fujikuroi*.

The culture filtrate from the Lt-1-3-3 fungal isolate was superior to that of *G. fujikuroi* in promoting plant growth, as the average shoot length of plants exposed to Lt-1-3-3 was 9.3 cm and the plant length was 20.1 cm in Waito-C seedlings (Fig. 2). These results are consistent with those of another study in which the endophytic fungus *P. citrinum*, isolated from the roots of *Ixeris repens*, was shown to promote the growth of Waito-C rice and *Atriplex gemelinii* seedlings [21].

To confirm the presence of gibberellins, microbial culture filtrates were screened to identify biologically active molecules. Waito-C rice seedlings were used for the detection of plant growth-promoting hormones in the culture filtrates of the fungal endophytes isolated from the roots of halophytes. Waito-C rice is a known dwarf rice cultivar with reduced biosynthesis of gibberellins. Waito-C seeds were treated with uniconazole as a gibberellin biosynthesis inhibitor. The Lt-1-3-3 fungal isolate, which has plant growth-promoting capacity (Fig. 2), was analyzed using Waito-C rice seedlings.

### Quantitative Analysis of Culture Filtrates of Lt-1-3-3 for the Presence of Gibberellins

**Fig. 3:**
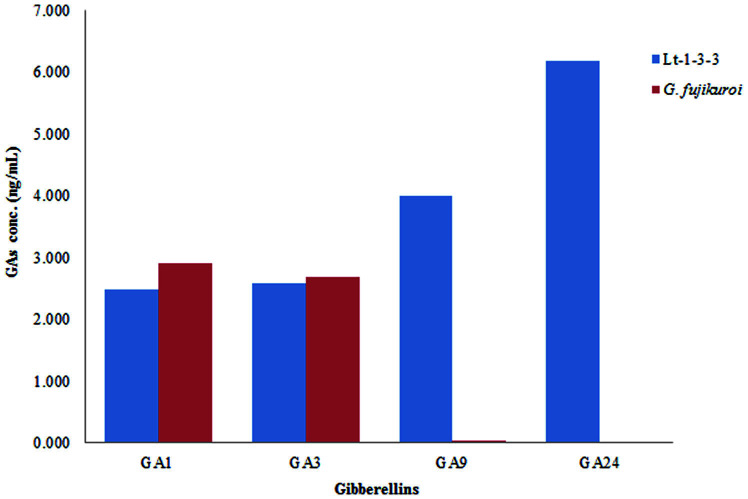
Gibberellins content of fungal culture filtrate of the Lt-1-3-3 isolate and wild type *Gibberella fujikuroi*. GC/MS SIM analysis of culture filtrate extracts from the Lt-1-3-3 fungal isolate detected two bioactive GAs. Lt-1-3-3 showed the presence of bioactivity of GA1, GA3, and other inactive GAs.

The plant growth-promoting activity of the culture filtrate of the fungus Lt-1-3-3 isolated from Waito-C rice germinals was confirmed. Analysis of gibberellin in this culture filtrate revealed the presence of GA_1_ (2.487 ng/ml), GA_3_ (2.592 ng/ml), GA_9_ (3.998 ng/ml), and GA_24_ (6.191 ng/ml). Fungal isolate Lt-1-3-3 produced significantly greater amounts of GA_9_ and GA_24_ than wild-type *G. fujikuroi*, which produced GA_1_ (2.909 ng/ml), GA_3_ (2.695 ng/ml), GA_9_ (0.043 ng/ml), and GA_24_ (0.026 ng/ml) in the present study (Fig. 3).

Including gibberellins, various secondary metabolites are known to be produced from fungal endophytes. The GC/MS with the SIM method makes it possible to analyze the complex mixtures and detect the compounds of different classes. Therefore, we used this technique for the cultural analysis of the Lt-1-3-3 fungal isolate. GC/MS SIM is helpful for the analysis of a number of compounds and is often used in plant experimentation. Due to its reliability, GC/MS SIM has been previously used in quantitative analyses of various plant hormones [22, 23].

In summary, 128 fungal isolates are isolated from 5 species of coastal plants. These fungal isolates were classified into 2 phyla, 6 classes, 12 orders, 17 families, and 31 genera. The dominant genus was *Fusarium* (class Sordariomycetes), followed by *Paraconiothyrium* and *Alternaria*. Based on the diversity analysis, the most diverse fungi were the endophytic fungi isolated from *S. maritima*. Culture filtrates from the 128 fungal isolates were assessed on Waito-C rice seedlings and the filtrate from fungal isolate Lt-1-3-3 exhibited excellent plant growth-promoting capacity. This isolate was confirmed to produce gibberellins GA_1_, GA_3_, GA_9_, and GA_24_. Using sequence homology, the Lt-1-3-3 isolate was identified as *G. intermedia*. This study provides basic data on the microbial resources present in plants from coastal salt marshes.

## Supplemental Materials

Supplementary data for this paper are available on-line only at http://jmb.or.kr.

## Figures and Tables

**Table 1:** Geographic coordinates and scientific names of plants native to the Seocheon salt marsh.

No	Scientific name	Code	Site of collection	Habitat
1	*Suaeda australis*	Sa	36° 3'18.55"N / 126°38'24.08"E	Halophytic
2	*Phragmites australis*	Pa	36° 2'45.24"N / 126°39'17.66"E	Halophytic
3	*Suaeda maritima*	Sm	36° 3'18.76"N / 126°38'26.84"E	Halophytic
4	*Suaeda glauca* Bunge	Su	36° 3'18.76"N / 126°38'22.32"E	Halophytic
5	*Limonium tetragonum*	Lt	36° 3'18.59"N / 126°38'23.60"E	Halophytic

**Table 2:** Endophytic fungi (128 isolates) isolated from plant species with scientific names, plant codes, taxa of fungal isolates, and number of isolates.

Scientific name of plant sample	Abbreviated plant name	Taxon of fungal strains	No. of isolates
*Suaeda australis*	Sa	12 genera, 11 species	23
*Phragmites australis*	Pa	11 genera, 8 species	15
*Suaeda maritima*	Sm	13 genera, 8 species	14
*Suaeda glauca* Bunge	Su	16 genera, 16 species	37
*Limonium tetragonum*	Lt	13 genera, 17 species	39

**Table 3:** Diversity indices and distribution of endophytic fungi isolated from plants native to the Seocheon salt marsh.

Fungal taxon	Sa	Pa	Sm	Su	Lt
*Alternaria*	2	4	1	3	4
*Aspergillus*				1	2
*Cladosporium*		2	2	2	1
*Clonostachys*		1			
*Colletotrichum*	2	1		2	2
*Curvularia*			1		1
*Epicoccum*			1		
*Exophiala*		1	1		
*Fusarium*	4	1	1	8	9
*Gibberella*					3
*Hypocrea*					2
*Lewia*			1		
*Macrophoma*	1		1		
*Microsphaeropsis*	1				
*Nectria*				1	
*Paraconiothyrium*	2			5	8
*Paraphaeosphaeria*	1				
*Paraphoma*				2	1
*Penicillium*	4	1	1	3	
*Pestalotiopsis*		1			4
*Phaeosphaeria*				1	
*Phoma*	3	1		1	
*Phomopsis*				1	
*Pilidium*				1	
*Plectosphaerella*	1				
*Pleospora*		1	1	1	
*Pseudozyma*	1	1	1		
*Rhizoctonia*			1		
*Stemphylium*					1
*Talaromyces*				3	
*Trichoderma*	1		1	2	1
*N*	23	15	14	37	39
*S*	12	11	13	16	13
Shannon diversity index (*H'*)	2.33	2.25	2.54	2.53	2.25
Simpson’s index of diversity (*1- D*)	0.93	0.93	0.99	0.92	0.89
Menhinick's index (*Dmn*)	2.50	2.84	3.47	2.63	2.08
Margalef 's index (*Dmg*)	3.51	3.69	4.55	4.15	3.28
Fisher's diversity (*α*)	10.12	18.60	88.78	10.71	6.83

Sa, *Suaeda australis*; Pa, *Phragmites australis*; Sm, *Suaeda maritima*; Su, *Suaeda glauca* Bunge; and Lt, *Limonium tetragonum*; *N*, total number of individuals in each samples; S; number of different genera in a sample.
