# Supplementary material for: Endophytic Fungi of Salt-Tolerant Plants: Diversity and Ability to Promote Plant Growth
Source: J Microbiol Biotechnol. 2021 Sep 15;31(11):1526–32. doi: 10.4014/jmb.2106.06007 (PMC9705876; doi:10.4014/jmb.2106.06007)
Supplement: Supplementary file 1 [file jmb-31-11-1526-supple.pdf]

J. Microbiol. Biotechnol.
https://doi.org/10.4014/jmb.2106.06007

Supplementary table 1.

Identification of fungal isolates from the roots of plant species.

Sample No.	Closely related fungal sequences	Similarity (%)	Accession No.
Sa-1-1-2	*Paraconiothyrium cyclothyrioides* strain YN(KC215138)	99	KP017930
Sa-1-3-1	*Macrophoma* sp. TXc4-6 (HQ262514)	100	KP017931
Sa-1-5-1	*Alternaria* sp. BJ35 (KJ702610)	100	KP017932
Sa-1-5-2	*Trichoderma* sp. IRB54b (KJ686346)	100	KP017933
Sa-1-6-1	*Fusarium oxysporum* strain HPA2 (KJ677253)	100	KP017934
Sa-1-7-2	*Penicillium canescens* strain CV0198 (JX140832)	100	KP017935
Sa-1-9-2	*Phoma* sp. JBARES02 (KC928322)	100	KP017936
Sa-1-9-3-2	*Microsphaeropsis arundinis* (JQ388915)	99	KP017937
Sa-1-10-3	*Alternaria* sp. BJ35 (KJ702610)	100	KP017938
Sa-2-3-1-2	*Paraconiothyrium cyclothyrioides* strain YN (KC215138)	100	KP017939
Sa-2-4-1	*Plectosphaerella* sp. MF-1 (AB520859)	100	KP017940
Sa-2-5-1	*Colletotrichum acutatum* strain 11E031 (KF717039)	100	KP017941
Sa-2-7-1	*Penicillium oxalicum* strain SY20-5 (KJ619622)	100	KP017942
Sa-2-10-1	*Fusarium oxysporum* strain HPA2 (KJ677253)	100	KP017943
Sa-3-3-1-1	*Fusarium incarnatum* strain FI-00602 (KJ572780)	99	KP017944
Sa-3-3-3	*Paraphaeosphaeria sporulosa* strain CBS (JX496227)	100	KP017945
Sa-3-4-1	*Penicillium brasilianum* (AB455514)	99	KP017946
Sa-3-7-1	*Pseudozyma* sp. CBS 10103 (AJ876488)	99	KP017947
Sa-3-7-2-1	*Penicillium* sp. CMV-2013f strain CV26 (JX140791)	99	KP017948
Sa-3-7-2-2	*Phoma* sp. P34E5 (JN207326)	99	KP017949
Sa-3-8-2	*Colletotrichum gloeosporioides* (EU294268)	99	KP017950
Sa-3-10-1-1	*Fusarium longipes* (KJ412506)	100	KP017951
Sa-3-10-2	*Phoma* sp. EF-40-2 (GU395514)	100	KP017952
Pa-1-2-2	*Pseudozyma* sp. CBS 10103 (AJ876488)	99	KP017953
Pa-1-6-1	*Cladosporium cladosporioides* isolate ZJ18 (KJ572146)	100	KP017954
Pa-1-7-1	*Alternaria* sp. BJ35 (KJ702610)	100	KP017955

Pa-1-7-2	*Cladosporium cladosporioides* strain GKF2 (KJ589558)	100	KP017956
Pa-1-8-3	*Alternaria* sp. DX-FOF7 (KF558883)	100	KP017957
Pa-2-3-4	*Penicillium decumbens* strain DHMJ02 (JN986754)	99	KP017958
Pa-2-5-1	*Pleospora bjoerlingii* (JX045842)	100	KP017959
Pa-2-5-2	*Exophiala oligosperma* (AB480204)	100	KP017960
Pa-2-6-1	*Alternaria* sp. BJ35 (KJ702610)	100	KP017961
Pa-3-1-1	*Fusarium longipes* (KJ412501)	100	KP017962
Pa-3-5-1	*Alternaria* sp. BJ35 (KJ702610)	100	KP017963
Pa-3-8-1	*Pestalotiopsis clavispora* strain F211b (JX045815)	100	KP017964
Pa-3-8-2	*Phoma* sp. JX1203 (KC203049)	100	KP017965
Pa-3-9-1	*Colletotrichum gloeosporioides* strain CG60 (KJ632430)	100	KP017966
Pa-3-9-3	*Clonostachys rosea* (KF736453)	100	KP017967
Sm-1-1-2	*Alternaria* sp. DX-FOF7 (KF558883)	100	KP017968
Sm-1-2-1	*Macrophoma* sp. TXc4-6 (HQ262514)	100	KP017969
Sm-1-3-1	*Lewia* sp. OUCMBI101191 (HQ914885)	100	KP017970
Sm-1-3-2	*Cladosporium cladosporioides* strain GKF2 (KJ589558)	100	KP017971
Sm-1-4-1	*Curvularia lunata* strain NF66 (KJ653820)	100	KP017972
Sm-1-6-2	*Rhizoctonia* sp. (JF519835)	100	KP017973
Sm-1-10-2	*Epicoccum nigrum* strain FKF2 (KJ589556)	100	KP017974
Sm-2-3-3	*Pseudozyma* sp. CBS 10103 (AJ876488)	99	KP017975
Sm-2-3-4	*Trichoderma harzianum* strain ML16-1 (KJ619615)	100	KP017976
Sm-2-10-2	*Exophiala oligosperma* (AB777520)	100	KP017977
Sm-3-3-1	*Cladosporium* sp. BJ45 (KJ702611)	100	KP017978
Sm-3-4-1	*Penicillium citrinum* strain NF7 (KJ653821)	100	KP017979
Sm-3-4-2	*Fusarium oxysporum* strain CY03 (KJ645965)	100	KP017980
Sm-3-10-1	*Pleospora bjoerlingii* (JX045842)	100	KP017981
Su-1-1-1	*Paraphoma* sp. BJ18 (KJ702586)	100	KP017982
Su-1-2-2	*Phomopsis* sp. 6309 (JN998102)	99	KP017983
Su-1-3-1	*Penicillium* sp. OY18307 (FJ571475)	100	KP017984
Su-1-4-1	*Colletotrichum gloeosporioides* strain CG60 (KJ632430)	100	KP017985
Su-1-4-3	*Talaromyces verruculosus* (JN676121)	100	KP017986
Su-1-6-1	*Pleospora bjoerlingii* (JX045842)	100	KP017987
Su-1-6-3	*Paraconiothyrium cyclothyrioides* strain YN (KC215138)	99	KP017988
Su-1-8-1	*Trichoderma harzianum* (KJ547595)	100	KP017989
Su-1-9-2	*Phoma* sp. Y19 (KJ572232)	100	KP017990

Su-1-9-4	*Fusarium proliferatum* strain PA3 (KJ701549)	100	KP017991
Su-2-1-2	*Trichoderma harzianum* strain ML16-1 (KJ619615)	100	KP017992
Su-2-2-1	*Paraconiothyrium cyclothyrioides* strain YN (KC215138)	99	KP017993
Su-2-2-3	*Penicillium rolfsii* strain SFCF20120912-04 (KF313082)	100	KP017994
Su-2-2-6	*Paraphoma* sp. BJ18 (KJ702586)	100	KP017995
Su-2-4-1	*Fusarium incarnatum* strain LS 03 (KJ721990)	100	KP017996
Su-2-4-2	*Nectria mauritiicola* (HF545314)	100	KP017997
Su-2-4-3	*Penicillium oxalicum* strain SY20-5 (KJ619622)	100	KP017998
Su-2-6-1	*Colletotrichum gloeosporioides* strain CG60 (KJ632430)	100	KP017999
Su-2-6-2	*Paraconiothyrium cyclothyrioides* strain YN (KC215138)	100	KP018000
Su-2-7-2	*Fusarium incarnatum* strain LS 03 (KJ721990)	100	KP018001
Su-2-9-1	*Cladosporium cladosporioides* strain GKF2 (KJ589558)	100	KP018002
Su-2-9-2	*Pilidium concavum* isolate PcSC (KF911079)	99	KP018003
Su-2-9-3	*Arthrinium* sp. E07 (KC867281)	100	KP018004
Su-2-9-3-1	*Alternaria* sp. BJ35 (KJ702610)	100	KP018005
Su-2-9-3-2	*Alternaria* sp. HT-M18-LS (KJ527010)	99	KP018006
Su-2-10-2	*Paraconiothyrium cyclothyrioides* strain YN (KC215138)	100	KP018007
Su-3-2-2	*Talaromyces verruculosus* (FR670340)	100	KP018008
Su-3-4-4	*Fusarium armeniacum* (KJ371100)	100	KP018009
Su-3-5-3	*Phaeosphaeria* sp. 1 TMS-2011 voucher (HQ631018)	100	KP018010
Su-3-6-1	*Paraconiothyrium cyclothyrioides* strain YN (KC215138)	100	KP018011
Su-3-6-3	*Aspergillus allahabadii* strain CBS 124597 (GQ342626)	100	KP018012
Su-3-6-4	*Cladosporium oxysporum* strain B2F2 (KJ589590)	100	KP018013
Su-3-7-1	*Fusarium incarnatum* strain LS 03 (KJ721990)	100	KP018014
Su-3-8-1	*Fusarium incarnatum* strain LS 03 (KJ721990)	100	KP018015
Su-3-8-2	*Fusarium armeniacum* (KJ371100)	100	KP018016
Su-3-10-1	*Fusarium oxysporum* strain HPA2 (KJ677253)	100	KP018017
Su-3-10-	*Talaromyces verruculosus* (JN676121)	100	KP018018

2-2

Lt-1-1-1	*Fusarium longipes* (HG423537)	99	KP018019
Lt-1-2-3	*Pestalotiopsis vismiae* isolate LH04Pv (JX305714)	100	KP018020
Lt-1-3-1	*Pestalotiopsis clavispora* strain P44 (JX045813)	100	KP018021
Lt-1-3-3	*Gibberella intermedia* strain Am3 (JX139607)	100	KP018022
Lt-1-4-2	*Gibberella intermedia* strain Am3 (JX139607)	100	KP018023
Lt-1-5-1	*Alternaria alternata* strain SR/I/90 (KJ767532)	100	KP018024
Lt-1-5-2	*Colletotrichum gloeosporioides* (KJ625631)	100	KP018025
Lt-1-7-1	*Trichoderma atroviride* strain ML29-3 (KJ619624)	100	KP018026
Lt-1-7-2	*Pestalotiopsis clavispora* strain P44 (JX045813)	100	KP018027
Lt-1-8-1	*Paraphoma* sp. BJ18 (KJ702586)	100	KP018028
Lt-1-9-1	*Paraconiothyrium cyclothyrioides* strain YN (KC215138)	100	KP018029
Lt-1-10-1	*Fusarium proliferatum* strain PA3 (KJ701549)	100	KP018030
Lt-2-1-1	*Fusarium incarnatum* strain FI-00602 (KJ572780)	100	KP018031
Lt-2-1-4	*Fusarium armeniacum* (KJ371100)	100	KP018032
Lt-2-1-5	*Colletotrichum gloeosporioides* (KJ625631)	100	KP018033
Lt-2-2-2	*Gibberella pulicaris* strain xsd08118 (FJ481029)	100	KP018034
Lt-2-2-3	*Fusarium caeruleum* (KC478528)	99	KP018035
Lt-2-2-4	*Aspergillus clavatus* strain AS-7 (EU515153)	100	KP018036
Lt-2-4-3	*Hypocrea* sp. SFCF20120803-50 (KF313111)	100	KP018037
Lt-2-5-4	*Paraconiothyrium cyclothyrioides* strain YN (KC215138)	100	KP018038
Lt-2-7-1	*Aspergillus* sp. BJ39 (KJ702608)	100	KP018039
Lt-2-7-2	*Paraconiothyrium cyclothyrioides* strain YN (KC215138)	99	KP018040
Lt-2-7-3	*Hypocrea* sp. SFCF20120803-50 (KF313111)	100	KP018041
Lt-2-8-1	*Alternaria* sp. BJ35 (KJ702610)	100	KP018042
Lt-2-8-2	*Alternaria alternata* strain SR/I/90 (KJ767532)	100	KP018043
Lt-2-8-3	*Cladosporium* sp. BJ45 (KJ702611)	100	KP018044
Lt-2-8-4	*Paraconiothyrium cyclothyrioides* strain YN (KC215138)	100	KP018045
Lt-2-9-1	*Stemphylium solani* strain CEF-772 (KF999031)	100	KP018046

Lt-3-1-2	*Paraconiothyrium cyclothyrioides* strain YN (KC215138)	100	KP018047
Lt-3-2-1	*Fusarium longipes* (HG423537)	99	KP018048
Lt-3-2-2	*Fusarium proliferatum* strain PA3 (KJ701549)	99	KP018049
Lt-3-3-1	*Alternaria alternata* strain SR/I/90 (KJ767532)	100	KP018050
Lt-3-4-1	*Pestalotiopsis* sp. 1 MJ-2014 (KJ572189)	99	KP018051
Lt-3-5-2	*Paraconiothyrium cyclothyrioides* strain YN (KC215138)	99	KP018052
Lt-3-5-3	*Paraconiothyrium cyclothyrioides* strain YN (KC215138)	99	KP018053
Lt-3-6-1	*Paraconiothyrium cyclothyrioides* strain YN (KC215138)	99	KP018054
Lt-3-7-1	*Fusarium oxysporum* strain CY03 (KJ645965)	99	KP018055
Lt-3-7-2	*Fusarium incarnatum* strain LS 03 (KJ721990)	99	KP018056
Lt-3-7-3	*Curvularia lunata* strain NF66 (KJ653820)	99	KP018057
